# Effect of Non-Cognitive Traits on Subjective Cognitive Decline among Black American adults

**DOI:** 10.1093/geroni/igaf122.2559

**Published:** 2025-12-31

**Authors:** Yolanda Jackson, Maria Clark, Sunkanmi Folorunsho, Obinna Odo, Candidus Nwakasi, Darlingtina Esiaka

**Affiliations:** University of Kentcuky, Lexington, Kentucky, United States; University of Kentucky, Lexington, Kentucky, United States; University of Nebraska-Lincoln, Lincoln, Nebraska, United States; Miami University, Oxford, Ohio, United States; University of Connecticut, Storrs, Connecticut, United States; University of Kentucky - College of Medicine, Lexington, Kentucky, United States

## Abstract

Research shows that subjective cognitive decline (SCD) is an early indicator of cognitive impairment, and it is associated with non-cognitive traits. Among Black Americans, non-cognitive traits such as grit and resilience have been found to influence their cognitive and emotional status. However, the role of grit and resilience in SCD among Black Americans remains understudied. This study examined the effects of grit and resilience on SCD among Black American men and women while controlling for age, socioeconomic status (SES), and marital status. Participants responded to standardized measures assessing SCD, grit, resilience, and demographic information. We performed multiple linear regression analyses using data from 242 participants and stratified by sex (N = 146 women; Mean age = 44.3). Among the Black American women, both grit and resilience were associated with SCD, with higher grit predicting higher SCD scores (β = 0.339, p < .001) and higher resilience predicting lower SCD scores (β = -0.234, p = .007). However, in men, only grit was associated SCD (β = 0.483, p < .001), with higher grit predicting higher SCD scores. Sex differences in the relationship between psychological resilience factors and SCD suggest that grit is a stronger predictor of SCD in both sexes, whereas resilience plays a protective role only in females. Understanding how non-cognitive traits impact SCD can help inform early interventions to help reduce cognitive decline risks in aging Black populations.

